# Physiology of Tasting

**Published:** 1865-04

**Authors:** Norton Folsom


### SELECTED.

### PHYSIOLOGY OF TASTING.

By Mr. NORTON FOLSOM.

When a substance to be tasted is placed in the mouth, we press it with the upper surface of the tongue against the palate, and thus force its particles in every direction. The saliva, poured in by its glands responsive to the stimulus, aids in dissolving and disseminating the particles over the mouth. When the substance reaches the fauces, and it is swallowed, a current of air escapes from the glottis, and carries any volatile portion to the posterior nares, where it is liable to affect the sense of smell. Plainly, therefore, in order to separate the two sensations, we must either shut off the cavity of the nose during the tasting, which can be done by most persons voluntarily by breathing through the mouth and applying the soft palate to the back of the pharynx, or we must interrupt the current of air through the nares, which can be done by holding the nose with the fingers.

We recognize two classes of impressions made by articles of food—one of *savors*, of which salt affords an example; the other of *flavors*, as that of vanilla. Most substances have both properties; thus a strawberry has an acid and a sweet taste, besides its own delicious flavor.

The distinction between these classes has not, indeed, been fully made by physiologists until of late; and still less has the fact been recognized, that *all flavors are perceived by the organs of smell only*, reducing the number of impressions which the organ of *taste* is capable of receiving four only, viz., Sweet, Sour, Salt and Bitter. This can, however, be easily and certainly demonstrated. Let the nose be closed by the fingers, or let the posterior nares be shut off by the soft palate, and a solution of vanilla be taken into the mouth and swallowed. It cannot be distinguished from water. Soup, nutmeg, cheese, pineapple, and asafœtida are alike entirely *flavorless* under similar conditions, though the ordinary sensibility of the mucous membrane, and the perception of the four savors above mentioned, may enable us to comprehend certain *other* qualities which distinguish these substances. The common practice of holding a child's nose while it swallows disagreeable medicine, has its origin in this peculiar relation of these two senses.

We have now to consider the exact locality of the sensations produced by these four classes of stimuli. Experiments have been tried by various physiologists with entirely different results, which may be attributed to want of care, and to not recognizing the fact that all *flavors* should be excluded from the investigation. All agree, however, in this—that, to be tasted a substance must be brought to the sensitive part *in solution*, inasmuch as insoluble substances have no taste.

In the experiments performed by the writer, solutions of white sugar, tartaric acid, common salt, and sulphate of quinine were carefully applied to various parts of the mouth and fauces by means of a camel's hair pencil, pains being taken that no excess of fluid should be used, which might diffuse itself over other parts than that directly under observation. The following results were uniformly obtained in six different individuals, they all being unaware of the substances used in each experiment:—

1st. The upper surface, tip, and edges of the tongue, as far back as to include the circumvallate papillæ, are the only parts concerned in the sense of taste; the hard and soft palate, tonsils, pharynx, lips, gums, and under surface of the tongue being entirely destitute of this sense.

2d. The circumvallate papillæ are by far the most sensitive portion of the organ. They perceive, at once, very minute quantities of any one of the four substances used, and are particularly sensitive to bitter. Irritating these papillæ by pressure, or placing a drop of cold water on them, excites decided sensations of bitterness.

3d. The central portion of the dorsum of the tongue, to within half an inch of the edge, is the least sensitive portion. Substances are distinguished with difficulty, or not at all, when applied to it.

4th. The edges and tip of the tongue are quite sensitive, the edges becoming less so as we come forward. They recognize all the four classes of substances. The tip detects bitter with great difficulty, but is particularly sensitive to sweet. A sweet sensation, sometimes mingled with sour or salt, is produced by gently tapping it with any insipid soft substance.

The tongue possesses *ordinary sensibility*, to a marked degree, especially at its tip, and in this way detects the size, shape, and texture of substances. It is in the same way that the qualities of pungency and astringency are perceived, which fact is proved by their being nearly imperceptible to the conjunctiva.—*Am. Drug. Cir. and Chem. Gaz.*

